# Providing Culturally Competent Care for COVID-19 Intensive Care Unit Delirium: A Case Report and Review

**DOI:** 10.7759/cureus.10867

**Published:** 2020-10-09

**Authors:** Christine M Lomiguen, Ivelys Rosete, Justin Chin

**Affiliations:** 1 Pathology, Lake Erie College of Osteopathic Medicine, Erie, USA; 2 Primary Care, Lake Erie College of Osteopathic Medicine, Erie, USA; 3 Medical Education, Lake Erie College of Osteopathic Medicine, Erie, USA; 4 Family Medicine, LifeLong Medical Care, Richmond, USA

**Keywords:** covid-19, icu, delirium, icu delirium, ventilator, cultural competency, filipino, covid 19, hospital

## Abstract

Coronavirus disease 2019 (COVID-19) was declared a pandemic by the World Health Organization in March 2020. Caused by the severe acute respiratory syndrome coronavirus 2 (SARS-CoV-2) virus, its high transmissibility required infected individuals to be placed in negative pressure isolation rooms when admitted to intensive care units (ICU). Studies have shown that limited social support can increase the risk of developing delirium during ICU stays. Minimal research exists on COVID-19-associated ICU delirium as hospitals and government organizations focus on combating equipment shortages and case surges. Here, we present the case of a 64-year-old Filipino male with COVID-19 ICU delirium status post-intubation and ventilation. His hospital course was complicated by the medical staff's assumption that the patient spoke Spanish and improved after being assigned a Tagalog-speaking nurse who facilitated family communication. This case highlights the importance of cultural competency and communication in the management of COVID-19 associated ICU delirium. In particular, Filipino cultural practices and their intersection with healthcare in the larger context of providing culturally competent care are highlighted. The use of culturally competent care serves to assure the use of appropriate services and reduces the occurrence of medical errors due to misunderstandings caused by differences in language or culture. Familial involvement is critical for ICU delirium; however, the COVID-19 pandemic has required healthcare providers to think beyond conventional means. The use of technology to virtually communicate with family also serves as a helpful tool to treat signs of delirium. As seen in this case, a lack of understanding of the Filipino culture resulted in assumptions on the part of the healthcare provider which led to the prolongation of delirium in a COVID-19 ICU patient, but the correct utilization of cultural competence helped the patient recover successfully.

## Introduction

Coronavirus disease 2019 (COVID-19), caused by the severe acute respiratory syndrome coronavirus 2 (SARS-CoV-2) virus, first emerged as a public health threat in December of 2019. Patients initially presented to hospitals with respiratory symptoms tied to food consumption stemming from a market in Wuhan, China [1]. According to the Centers for Disease Control and Prevention, the virus has continued to spread throughout the United States with a doubling time of approximately 6.5 days and a basic reproductive number of approximately 2.5 worldwide [2]. The rapid transmission rate of the virus has placed enormous pressure on researchers and healthcare systems worldwide to develop containment strategies and treatment options. As of June 7th, 2020, there have been 109,479 deaths caused by COVID-19 in the United States [3]. With variable enforcement and public adherence to social distancing and other containment methods, coupled with the lack of a cure or vaccine, infections and deaths attributed to COVID-19 are expected to continue escalating in the foreseeable future.

Patients with COVID-19 can present along a wide spectrum, ranging from asymptomatic carriers to acute respiratory failure. Regardless of the physical manifestation, patients with COVID-19 can infect others through aerosolization of virus-filled droplets, fomite contamination, and interaction with other bodily fluids [1,4]. The incubation period can last from two to 14 days, in which constitutional and respiratory symptoms such as fever, cough, and dyspnea may occur [4]. Patients who are over 50 years of age and those with underlying chronic medical conditions are more likely to experience severe COVID-19 symptoms, such as hypoxemia and respiratory distress [5]. Severe COVID-19 symptoms typically result in hospitalization and mechanical ventilation in an intensive care unit (ICU). Infection control and public health guidelines for COVID-19 have necessitated hospitals to restrict visitation and supportive services, requiring patients to convalesce in isolation [4,5].

Delirium is defined as a disturbance in mental abilities that results in severe confusion and reduced awareness of one's surroundings [6]. Although the mechanism is still largely unknown, ICU patients have been shown to be at a higher risk for developing delirium. ICU delirium is associated with poorer health outcomes as patients have a prolonged hospital course and duration of mechanical ventilation, greater rates of self-extubation, and higher overall mortality and morbidity [7]. Supportive care through family and other sources has been recognized as a protective factor for the development and treatment of ICU delirium [6,7]. COVID-19 patients, in particular, are at risk for developing ICU delirium due to isolation, family visitation restrictions, and staffing shortages. With the limitations on family and outside support, the responsibility of decreasing the risk and ameliorating the effects of COVID-19 ICU delirium falls upon the medical team. Culturally competent care has been shown to improve health outcomes and is critical to fostering an inclusive environment [8,9].

Here we present a case of a 64-year-old Filipino male with COVID-19 associated ICU delirium and the role that culturally competent care plays in the healing process during a global pandemic.

## Case presentation

Mr. L is a 64-year-old Filipino male who presented to the emergency department (ED) with a one-day history of shortness of breath, which did not improve on rest. Past medical history was significant for hypertension, hyperlipidemia, diabetes mellitus type 2, asthma, obstructive sleep apnea, and obesity. His social history was significant for being a former smoker with 10 pack years and having quit 30 years ago. Prior to ED assessment, the patient reported a five-day history of fever (maximum temperature of 104°F, with no improvement on acetaminophen) and four days of non-productive cough, body aches, and diarrhea. The patient was an essential worker as a chemical plant operator who interacted with many potential sick contacts. 

Vitals on admission revealed an oxygen saturation of 87% and temperature of 102°F. The patient was tachypneic and tachycardic, with respirations of 20 breaths per minute and heart rate of 110 beats per minute. Chest x-ray revealed diffuse bilateral infiltrates and he was admitted due to progressive oxygen desaturation. COVID-19 nasal swab with reverse transcriptase polymerase chain reaction (rtPCR) was done on hospital day two, which returned positive on hospital day four. During this time, the patient began to deteriorate, progressing from using a nasal cannula and non-rebreather mask to requiring intubation and mechanical ventilation. Various combinations of fentanyl, propofol, and midazolam were used for comfort in addition to a trial of hydroxychloroquine. He was extubated on hospital day 23 after showing several days of marked improvement in oxygenation.

Upon extubation on hospital day 23, the patient exhibited signs of agitation, anxiety, and confusion, manifested by disordered speech, disorientation to place and time, and attempts to remove intravenous (IV) lines and monitors. This continued until hospital day 30 in which the patient was found collapsed on the floor and laughing hysterically. Physical restraints and pharmacological mixtures of midazolam, dexmedetomidine, and lorazepam were used as needed. Limited intervention occurred during this period due to staffing shortages and lack of resources. During a telephone update with family members on hospital day 32, the health care team disclosed that nursing had been speaking to the patient in Spanish to encourage a more familiar atmosphere using their native language instead of English. Due to the rapid patient decline and expedited triage during admission, language preference and other identifiers were omitted from the chart. With this revelation, the family member informed that the patient did not speak Spanish, but rather was fluent in English and Tagalog (one of the languages of the Philippines). In the following days, a Tagalog speaking nurse or nursing assistant was assigned to the patient to facilitate cultural communication regarding the patient’s health care and wishes. During this time, the patient was able to emphasize the importance of family and obtained a tablet to video call his wife and daughter, which resulted in a decline of delirium symptoms.

The patient was downgraded to the medical floor on hospital day 33 and discharged to an acute rehabilitation facility on hospital day 35. On first assessment with cognitive rehabilitation therapy, immediate and delayed verbal recall was impaired, but showed gradual improvement. The patient was discharged from the acute rehabilitation facility after 10 days with full recovery at home.

## Discussion

The COVID-19 pandemic has created a perfect storm of conditions that promote ICU delirium. Twenty percent of COVID-19 positive patients require hospitalization and up to a quarter will require admission to an ICU, which represents five to 11% of the total infected population [10]. Early studies have shown that 20-30% of COVID-19 patients will present with or develop delirium during the course of their hospitalization, with a rate of 60-70% in cases of severe illness at all ages. Of those admitted to the ICU that are COVID-19 positive, 73.6% of these patients experience delirium that persists for approximately one week [10,11]. The diagnosis of ICU delirium and COVID-19 ICU delirium are nearly identical and are made based on the acute onset of disturbance of attention and awareness, impaired cognition, agitation, and a sleep-wake cycle dysregulation in context of medical illness [11]. Treatment of ICU delirium typically involves the patient’s family and support groups to create a familiar and inviting environment to gently reorient patients to their surroundings. These comforts, however, have been eliminated during the COVID-19 pandemic due to concerns for community level transmission and spread.

**Figure 1:**
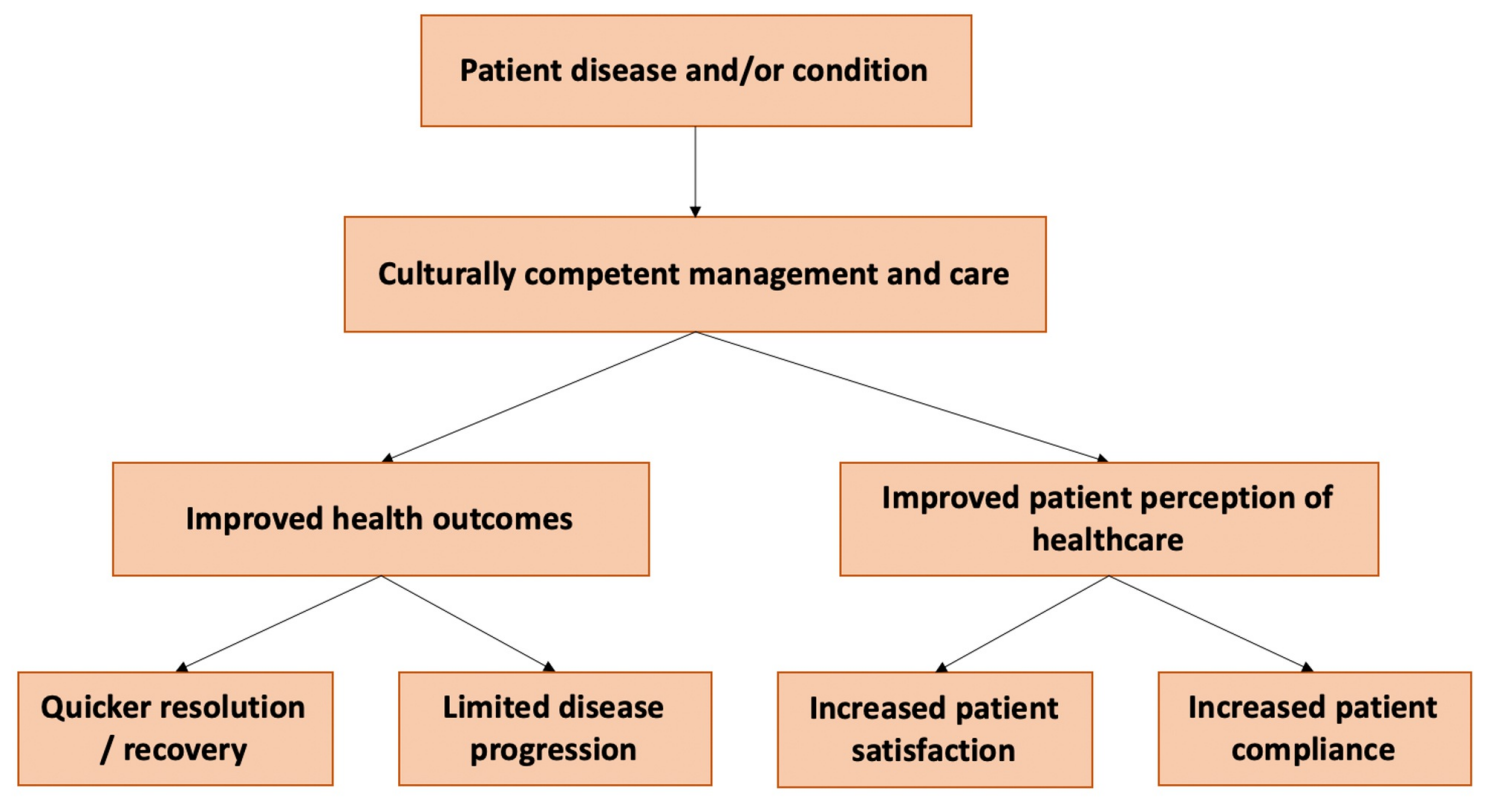
Schematic diagram of ideal culturally competent care and its outcomes.

The Centers for Disease Control and Prevention, along with state, local, and hospital administrations, have promulgated guidelines regarding management of visits to healthcare facilities in the context of the COVID-19 pandemic [12]. The rationale for these limitations is two-fold: prevention of spread from the community to the hospital (and vice versa) as well as safeguarding vulnerable populations in hospital settings. As COVID-19 patients are considered highly contagious, ICUs across the United States have implemented isolation protocols and negative pressure containment rooms to prevent community spread [13]. While important from an infection control and public health perspective, COVID-19 ICU patients have increasingly developed delirium status post extubation and mechanical ventilation weaning [10,11]. Surrounded by faceless, masked personnel covered in protective equipment and a lack of family and supportive services that usually act as patient advocates and assistants during episodes of delirium, recovery from COVID-19 ICU delirium has been found to take longer than classical ICU delirium [7]. As seen in this case, the intense social isolation and increased use of both physical and chemical restraints for fear of transmission of COVID-19 can result in the exacerbation of delirium and prolongation of its course, resulting in poorer outcomes and accelerated mortality. This is in comparison to the ideal patient care with cultural competency (Figure 1). 

Limitations on providing humanistic care to COVID-19 ICU patients require healthcare workers to utilize alternative methods to minimize and treat delirium. Culturally competent care is defined as the ability and practice of healthcare professionals to respect the diversity in patients and acknowledges the role of cultural factors (language, ethnic beliefs, attitudes, and behaviors) in how patients interact with the healthcare system [14]. In the case of a COVID-19 ICU patient, feelings of isolation can lead to a worsening of well-being, depressive symptoms, anxiety, and anger, which can hasten the development of delirium [7]. Engaging patients through a familiar language, soliciting their beliefs and customs, incorporating family input, and having open body language are ways how culturally competent care is traditionally incorporated. Many of these methods have not been possible during the COVID-19 pandemic. In the aforementioned case, the staff attempted to apply culturally competent care, however, preconceived assumptions regarding the patient’s ethnicity and language hindered advancement of care. In this case, coping mechanisms such as “pakikisama”-conceding to the wishes of the collective and “bahala na”-fatalistic resignation in which the patient’s fate is up to God, are important to consider in working with Filipino patients [15-17]. In interacting with healthcare workers, personalism or “hindi ibang tao" - one of us/"ibang tao"-not one of us may dictate the level of trust and openness that a Filipino patient displays [18]. With Mr. L, having been spoken to in a foreign language (Spanish), this could have contributed to the conclusion that the staff was ibang tao or "not one us" and worsened his delirium. Improving cultural competence in medical staff can help to reduce biases, which in turn may improve patient outcomes and the patient experience [19].

The best known non-pharmacological solution to combat delirium has been to promote communication between the patient and family [20]. Unfortunately, the limitation on visitation makes personal, physical contact with loved ones impossible and further increases their risk of entering a state of delirium. Culturally, family is paramount in the Filipino community, with filial piety and intergenerational decision-making being key elements that intersect with healthcare decisions [15-17]. Other non-pharmacological, non-culture based solutions that are recommended include minimizing disruptions at nighttime to promote proper sleep, providing cognitive stimulation, providing physical mobilization and encouraging the use of family photos, phone calls, and virtual visits [11]. As seen with Mr. L, the use of a hospital tablet to communicate with family members virtually can be used as a beneficial tool to treat signs of delirium. To prevent hindering a COVID-19 patient’s recovery, it would be ideal for medical staff to call family members, if the patient is unable to participate, to discuss a plan of care and seek a mutual agreement. Overall, the correct use of cultural competence by healthcare professionals is a crucial tactic to reduce some of the risk factors associated with delirium and is the best approach to provide patient-centered care during these difficult times.

## Conclusions

When working with COVID-19 patients of a different culture throughout the pandemic, it is important for healthcare providers to accurately apply cultural competence to reduce the risk of, and ameliorate, ICU delirium. The use of culturally competent care serves to assure the use of appropriate services and reduces the occurrence of medical errors due to misunderstandings caused by differences in language or culture. Familial involvement is critical for ICU delirium, however the COVID-19 pandemic has required healthcare providers to think beyond conventional means. The use of technology to virtually communicate with family also serves as a helpful tool to treat signs of delirium. As seen in this case, a lack of understanding of the Filipino culture resulted in assumptions on the part of the healthcare provider which led to the prolongation of delirium in a COVID-19 ICU patient, but the correct utilization of cultural competence helped the patient recover successfully. Similar considerations can be applied to other patients with COVID-19 ICU delirium as culture plays an important role in how patients understand and interface with the healthcare system.
